# Supplementary material for: Steady Moderate Exercise Confers Resilience Against Neurodegeneration and Neuroinflammation in a Mouse Model of Parkinson’s Disease
Source: Int J Mol Sci. 2025 Jan 28;26(3):1146. doi: 10.3390/ijms26031146 (PMC11818830; doi:10.3390/ijms26031146)
Supplement: Supplementary file 1 [file ijms-26-01146-s001.zip › ijms-3410016-supplementary.pdf]

Table S1. A list of the antibodies used in this study, their suppliers and the dilutions used.

**Primary antibody**	**Supplier**	**Primary antibody dilution**	**Secondary antibody**	**Supplier**	**Secondary antibody dilution**
Anti-tyrosine hydroxylase (TH, Cat #AB152)	Merck	1 : 1000	Biotinylated Goat Anti-Rabbit IgG Antibody (Cat #BA-1000)	Vector Laboratories	1 : 200
Anti-dopamine receptor D2 (DRD2, Cat #PA5-115142)	Invitrogen	1 : 200	Biotinylated Goat Anti-Rabbit IgG Antibody (Cat #BA-1000)	Vector Laboratories	1 : 200
Anti-glial cell line-derived neurotrophic factor (GDNF, Cat #sc-328)	Santa Cruz Biotechnology	1 : 500	Biotinylated Goat Anti-Rabbit IgG Antibody (Cat #BA-1000)	Vector Laboratories	1 : 200
Anti-brain-derived neurotrophic factor (BDNF, Cat #sc-20981)	Santa Cruz Biotechnology	1 : 500	Biotinylated Goat Anti-Rabbit IgG Antibody (Cat #BA-1000)	Vector Laboratories	1 : 200
Anti-Integrin subunit alpha M (CD11b, Cat #MCA711G)	Bio-Rad	1 : 200	Goat anti-Rat IgG (H+L) Cross-Adsorbed Antibody, Alexa Fluor 488 (Cat #A-11006)	ThermoFisher	1 : 1000
Anti-glial fibrillary acidic protein (GFAP, Cat #Z0334)	Dako	1 : 1000	F(ab')2-Goat anti-Rabbit IgG (H + L) Cross-Adsorbed Antibody, Alexa Fluor 488 (Cat #A-11070)	ThermoFisher	1 : 1000
Anti-ionized calcium binding adaptor molecule 1b (Iba-1b, Cat #ab5076)	Abcam	1 : 500	Rabbit Anti-Goat IgG Antibody, HRP conjugate (Cat #AP106P)	Merck	1 : 500

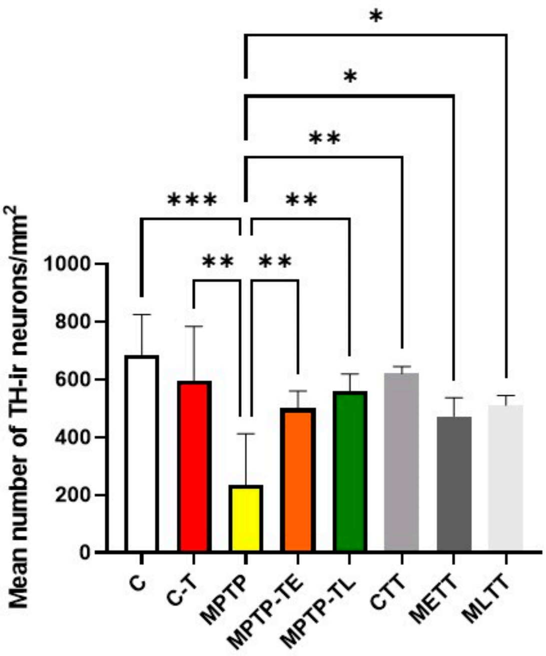

Figure S1. Mean number of TH-immunoreactive neurons in the substantia nigra pars compacta (SNpc) of control mice (C), moderate intensity trained control mice (C-T), untrained mice with induced Parkinsonism (MPTP), mice with induced Parkinsonism which underwent early-onset moderate intensity training (MPTP-TE), mice with induced Parkinsonism which underwent late-onset moderate intensity training (MPTP-TL), high intensity trained control mice (CTT), mice with induced Parkinsonism which underwent early-onset high intensity training (METT), mice with induced Parkinsonism which underwent late-onset high intensity training (MLTT).

For TH-immunoreactive neurons in the SNpc, a one-way ANOVA confirmed a significant main effect of Group ($F (7, 23) = 6{,}043$; $p=0.0004$). MPTP mice presented strongly reduced TH labeling relative to all control groups. While the mean number of the TH-ir neurons was reduced in MPTP relative to control groups, this reduction has been alleviated when physical exercise was administered. Both early-onset and late-onset treadmill training favorably affected the preservation of the dopaminergic phenotype of neurons in the SNpc. The moderate and high intensity training counteracted the TH-ir neurons reduction to similar degree. Summing up, neurotoxin administration reduced the number of TH-ir neurons while exercise counteracted this reduction, regardless of when this exercise was applied and what intensity was used. Statistical significance between the groups was calculated using the Newman–Keuls test (* $p < 0.05$, ** $p < 0.01$, *** $p < 0.001$).

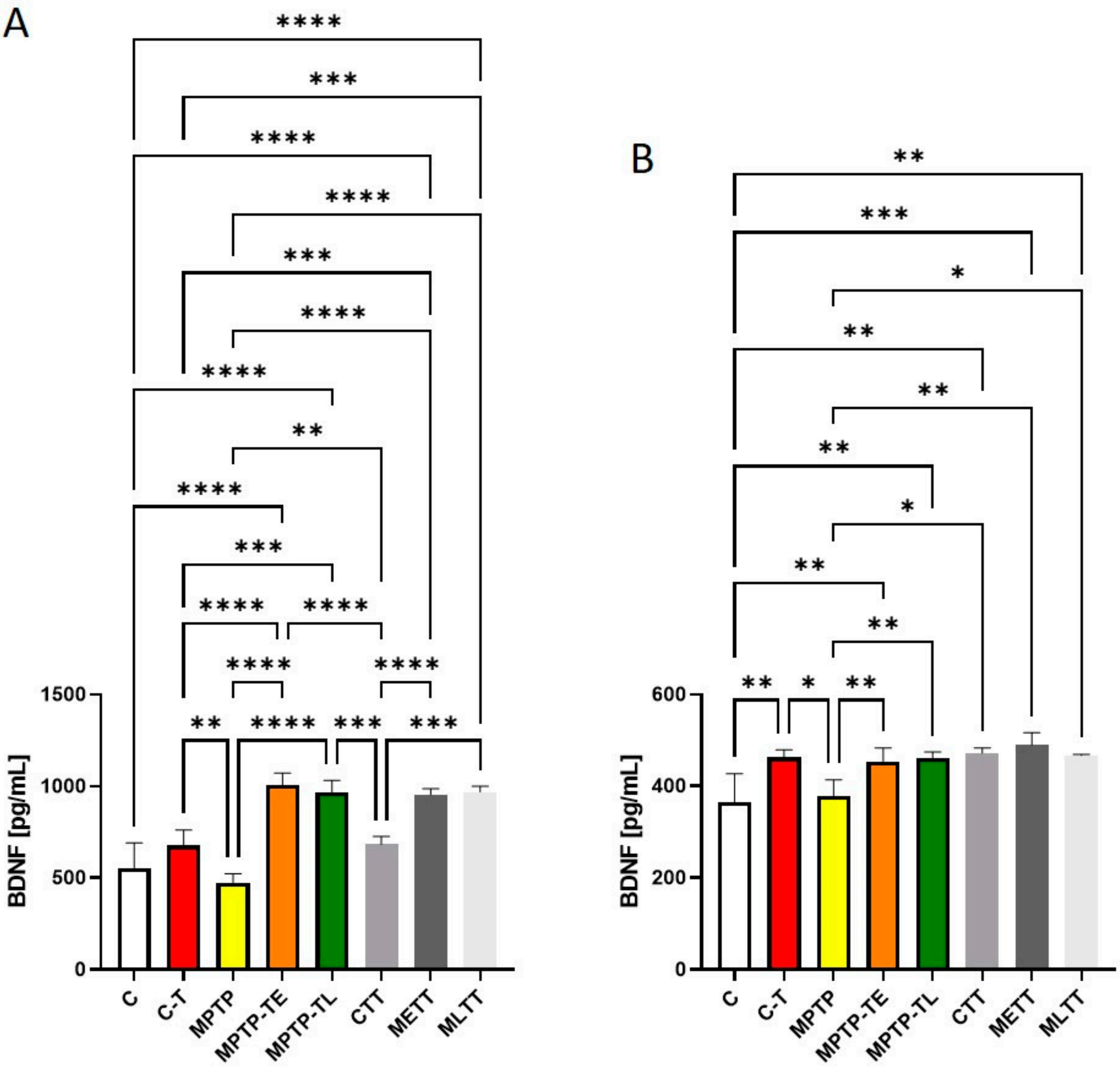

Figure S2. Quantitative analysis of the levels of brain derived neurotrophic factor (BDNF) by ELISA in the midbrain (**A**) and in the striatum (**B**) of control mice (C), moderate intensity trained control mice (C-T), untrained mice with induced Parkinsonism (MPTP), mice with induced Parkinsonism which underwent early-onset moderate intensity training (MPTP-TE), mice with induced Parkinsonism which underwent late-onset moderate intensity training (MPTP-TL), high intensity trained control mice (CTT), mice with induced Parkinsonism which underwent early-onset high intensity training (METT), mice with induced Parkinsonism which underwent late-onset high intensity training (MLTT). Values are presented as the mean ± SD with scatter plots of individual data.

(A) Quantitative analysis of the BDNF level in the midbrain with one-way ANOVA confirmed a significant main effect of Group (F (7, 24) = 27,36; p<0.0001). Untrained MPTP mice did not show strongly reduced BDNF level compared to control group (C), whereas BDNF levels were elevated not significantly when physical exercise was administered in trained controls (C-T, CTT) and reached significantly higher level when training was combined with MPTP treatment (MPTP-TE, MPTP-TL, METT, MLTT). It can be concluded that BDNF upregulation reached similar level regardless of training intensity and also irrespective when training was commenced. **(B)** Quantitative analysis of the BDNF level in the striatum with one-way ANOVA yielded the main effects of Group (F (7, 24) = 6,877; p=0.0002). Similarly, as observed in the midbrain, untrained MPTP mice did

not show strongly reduced BDNF level compared to control groups (C), whereas BDNF levels were similarly elevated when physical exercise was administered in trained controls (C-T, CTT) and when training was combined with MPTP treatment (MPTP-TE, MPTP-TL, METT, MLTT). Statistical significance between the groups was calculated using the Newman–Keuls test (* $p < 0.05$, ** $p < 0.01$, *** $p < 0.001$, **** $p < 0.0001$).

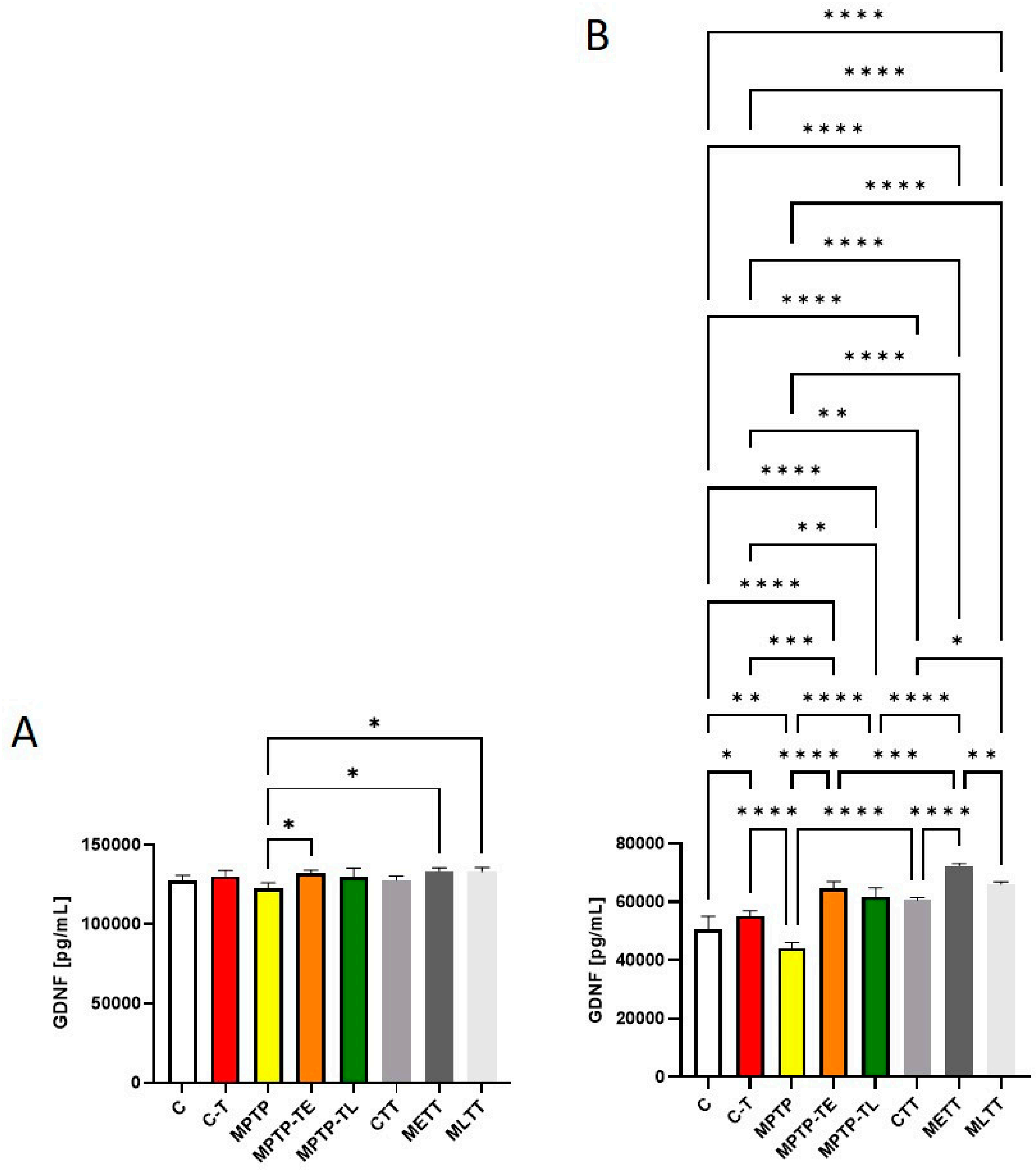

Figure S3. Quantitative analysis of the levels of Glial derived neurotrophic factor (GDNF) by ELISA in the midbrain (**A**) and in the striatum (**B**) of control mice (C), moderate intensity trained control mice (C-T), untrained mice with induced Parkinsonism (MPTP), mice with induced Parkinsonism which underwent early-onset moderate intensity training (MPTP-TE), mice with induced Parkinsonism which underwent late-onset moderate intensity training (MPTP-TL), high intensity trained control mice (CTT), mice with induced Parkinsonism which underwent early-onset high intensity training (METT), mice with induced Parkinsonism which underwent late-onset high intensity training (MLTT). Values are presented as the mean ± SD with scatter plots of individual data.

(A) Quantitative analysis of the GDNF level in the midbrain with one-way ANOVA showed a significant main effect of Group (F (7, 24) = 2,951; p=0.0223). MPTP mice showed not significantly reduced GDNF level compared to untrained (C) and trained (C-T, CTT) control groups. However, GDNF level was

significantly lower in MPTP group when compared to MPTP groups trained with early-onset moderate intensity and with early- and late-onset high intensity one. Thus, BDNF level in MPTP-trained groups reached the same level as observed in controls. **(B)** Quantitative analysis of the GDNF level in the striatum with one-way ANOVA yielded the main effects of Group (F (7, 24) = 51,36; $p<0.0001$)). Unlike in the midbrain, untrained MPTP mice showed significantly reduced GDNF level in the striatum compared to untrained (C) and trained (C-T, CTT) control groups. High intensity training caused greater elevation of GDNF level than moderate one; GDNF level in CTT, METT was significantly higher than in C-T, MPTP-TE, respectively. The highest GDNF upregulation was noted in early-onset high intensity training (METT). Statistical significance between the groups was calculated using the Newman–Keuls test (* $p < 0.05$, ** $p < 0.01$, *** $p < 0.001$, **** $p < 0.0001$).
